# Non-synchronization of lattice and carrier temperatures in light-emitting diodes

**DOI:** 10.1038/srep19539

**Published:** 2016-01-20

**Authors:** Jihong Zhang, Tienmo Shih, Yijun Lu, Holger Merlitz, Richard Ru-Gin Chang, Zhong Chen

**Affiliations:** 1 Department of Electronic Science, Fujian Engineering Research Center for Solid-state Lighting, State Key Laboratory for Physical Chemistry of Solid Surfaces, Xiamen University, Xiamen 361005, China; 2 Department of Physics, Xiamen University, Xiamen 361005, China; 3 Institute for Complex Adaptive Matter, University of California, Davis, CA 95616 USA; 4 Leibniz Institute for Polymer Research, Dresden, Germany; 5 Shineraytek Optoelectronics Co., Shanghai 201300, China

## Abstract

Pulse implementation or switching-off (PISO) of electrical currents has become a common operation in junction-temperature (*T*_*j*_) measurements for semiconductor devices since 2004. Here we have experimentally discovered a substantial discrepancy between *T*_*j*_ values with, and without, PISO (e.g., 36.8 °C versus 76.5 °C above the ambient temperature at 25.0 °C). Our research indicates that methods associated with PISO are flawed due to non-synchronization of lattice temperatures and carrier temperatures in transient states. To scrutinize this discrepancy, we propose a lattice-inertia thermal anchoring mechanism that (1) explains the cause of this discrepancy, (2) helps to develop a remedy to eliminate this discrepancy by identifying three transient phases, (3) has been applied to establishing an original, accurate, and noninvasive technique for light-emitting diodes to measure *T*_*j*_ in the absence of PISO. Our finding may pave the foundation for LED communities to further establish reliable junction-temperature measurements based on the identified mechanism.

In designing light-emitting diodes (LEDs)^1^,^2^,^3^ that emit the light via recombination of holes and electrons and waste thermal energy through lattice vibration, we desire to extract photons (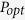), must supply electrons (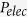), and dislike phonons (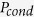) (Fig. 1a). In turn, characteristics of photons, electrons, and phonons are strongly associated with the temperature at the junction interface (*T*_*j*_) between n-type and p-type semiconductors^4^,^5^,^6^,^7^. It is currently a challenge to accurately measure LED junction temperatures^8^,^9^,^10^,^11^ (*T*_*j*_) under conditions of large currents^12^,^13^,^14^,^15^. The primary reason arises because LED chips are usually sealed, thus prohibiting direct contacts. Presently, the work related to pulse implementation or switching-off (PISO, Fig. 1b) has populated the literature in semiconductor areas, including forward voltages^16^,^17^, peak energy^18^,^19^, reverse currents^20^, and low forward currents^21^. Although these methods are capable of facing the challenge mentioned above, the discrepancy between results obtained with, and without, PISO has been found to be substantial.

In our laboratory, we have adopted both the forward voltage method (FVM, Fig. 1b) and confocal Raman spectroscopy (CRS, Fig. 1c). Using the former, we first obtain the steady-state linear relationship between *T*_*j*_ (inset of Fig. 2a), controlled by the heat sink at 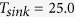 °C, and the forward voltage 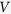 at 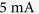 with negligible thermal power input. Then we light the LED sample (e. g. blue InGaN/GaN) under a large steady-state current (e. g. 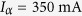). Instantaneously, this current is switched down to 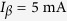 by the FVM instrument named T3ster (MicRed. Inc., Hungary), and the forward voltage is recorded. Utilizing the linear relationship at 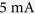, we deduce the desired *T*_*j*_ to be 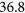 °C under 350 mA (Fig. 2a, time in logarithmic scale). Alternatively, when using CRS^22^,^23^,^24^, which excludes PISO, we obtain *T*_*j*_ to be 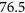 °C based on the peak location of Raman shift (Fig. 2b,c). Peaks of Raman-light-beam intensity shift to the left as *T*_*j*_ increases by an increment of 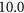 °C, whereas the peak at 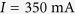 and 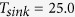 °C (the ▲ curve) is located at 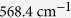 (Fig. 2d). This trend clearly suggests that *T*_*j*_ must be at least higher than approximately 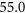 °C + 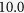 °C. Had *T*_*j*_ been lower than 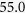 °C, as measured by FVM, the peak should have been located between 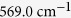 and 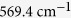. Relative to the ambient temperature at 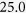 °C, the discrepancy amounts to 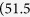 °C − 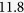 °C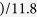 °C = 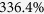.

To scrutinize this puzzling difference, we have additionally used thermocouples (TC)^25^ and a thermal imager (TI)^26^, which receive direct thermal signals from samples, and have obtained 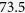 °C and 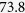 °C (Fig. 2e–g), respectively (Supplementary S1). Nine exposed LEDs (1-W each) were further selected for conformations, including three blue InGaN/GaN (B1#, B2#, B3#), three green InGaN/GaN (G1#,G2#, G3#) and three red AlGaInP (R1#, R2#, R3#), also leading to substantial discrepancies (Supplementary S2). Finally, we propose the following mechanism to explain this discrepancy, and further develop an independent method that requires neither PISO nor intrusive contacts, and utilizes Shockley equation for diodes as well as the principle of thermal anchoring (to be described below).

## Lattice-inertia thermal anchoring (LITA)

**Figure 1:**
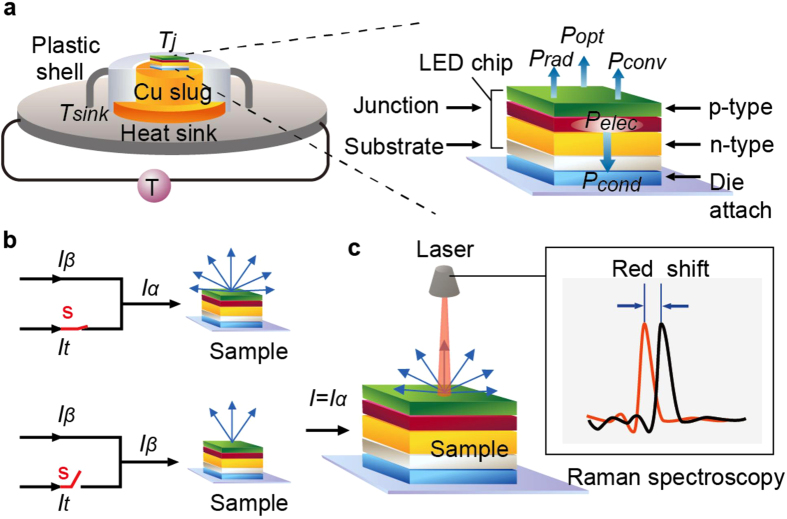
Experimental set-ups for forward voltage method (FVM). (**a**) The LED sample consists of layers including LED chip, die attach, and copper slug. In steady state, the influx IV should equal out fluxes including 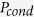, 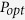, 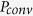, and 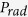 (cond = conduction; opt = optical; conv = convection; rad = radiation). (**b**) Pulse-implementation or switching-off (PISO) of currents for FVM. 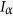 = current at the steady state, e. g., 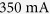; 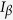 = a small fraction of 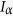 to stay on, e. g., 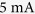; 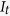 = the major portion of 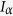 to be switched off. The subscript ‘
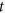
’ denotes ‘thermal’, suggesting that the current generates the thermal power. (**c**) Confocal Raman spectroscopy (CRS). The LED sample is mounted on a heat sink, and is lit by a current source. The peak of Raman shift has moved leftward minutely when temperatures of samples increase.

Consider the electron transport inside a doped semiconductor undergoing three transient phases: (1) PISO phase from state 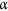 to state 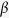, (2) non-synchronization phase from state 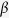 to state 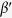 (a delayed state replacing state 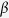), (3) relaxation phase from state 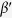 to 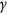 (steady state). The electron velocity at steady state 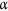 should equal the vector sum of the thermally-diffusive velocity and the drift velocity. After algebra, we can prove that the kinetic energy of electrons at state 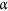 is greater than the counterpart at state 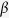 (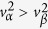) (Supplementary S3), partly because the drift component diminishes upon PISO. Electrons with small drift velocities descend to combine with holes in the valence band, reducing potential energies relative to their nucleuses. Consequently, the carrier temperature (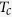)^27^,^28^,^29^,^30^,^31^ decreases from state 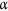 to state 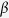. Next, there exist two types of external inputs, electrical power and thermal power that influence both 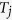 and 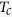 (Fig. 3a). For the former which drives the electron transport, 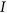 and 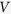 change instantaneously after PISO, exerting impacts on the electrical field, which subsequently causes reductions of 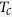 or carrier potential energy. Because of lattice inertia 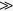 carrier inertia and the occurrence of PISO, we can conclude that 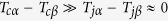. Consider a practical example, in which the electrical current of 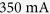 (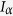) is instantaneously switched down to 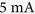 (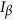) within approximately 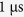, along with a voltage reduction from 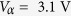 to 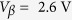 (Fig. 3b). Complicated phenomena, including re-thermalization, radiative recombination, non-radiative Auger, and non-radiative Shockley-Reed-Hall deep-level recombinations, diminish as time elapses within the sample. Let us calculate dimensionless percentage changes of 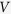, 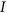, and 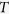 as 
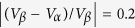
, 
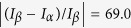
, 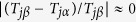, and 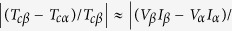

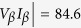
. These changes imply that 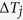 and 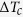 differ substantially, leading to the chaotic nature of state 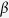 and the difficulty of determining 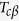 and 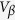 accurately. Hence, if possible, we should avoid utilizing data that belong to the uncertain 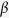 state, completely dismiss 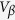 that plays the primary role of the discrepancy-inducing culprit, and proceed to cool down the sample further till state 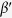. From state 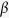 to state 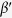, *T*_*j*_ is primarily influenced by the external cooling macroscopically or phonon propagation and lattice vibrations microscopically (Fig. 3a). By contrast, electrons continue to descend from higher to lower energy levels, but the descending distance becomes smaller than that from state 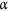 to state 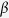. This loss in kinetic and potential energies is converted into the outgoing Planck radiation at larger wavelengths. Even though the magnitude of Planck radiation appears small, it is the primary macroscopic thermal cooling mechanism for 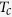. According to the principle of energy conservation over a control volume containing carriers only, we obtain


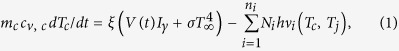


where 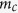 is the effective mass of carriers, 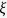 the percentage of external inputs that are converted into the kinetic energy of carriers, 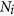 the number of electrons emitting at the frequency of 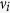 and 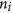 the number of energy states. Likewise, the lattice also proceeds to cool down due to slower oscillations of the heat-sink lattice. Based on the principle of energy conservation over the control volume containing the lattice only, we also obtain


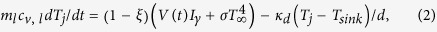


where 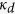 is the overall thermal conductivity of layers, 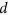 the thickness of layers and the subscript ‘
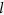
’ denotes ‘lattice’. Equations (1) and (2) suggest that 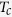 and 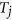 are governed by different thermal-cooling mechanisms as well as by their appreciably-different thermal inertias (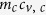 and 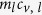), dictating that they must vary at different paces. At steady states, the inter-relationship among 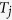, 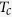 and the voltage (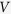) must be unique at fixed currents and sink temperatures. Therefore, it is nonrigorous for FVM to apply this inter-relationship to situations when 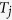 and 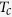 vary at different paces. In short, for given LED types and constant small (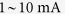
for 1-W high power LEDs) currents, FVM asserts that, even in transient states, 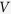 is a linear function of 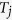 only. In the proposed study, 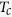 → kinetic energy of carriers → different thermal-equilibrium states → Fermi levels → external voltages, where “→” denotes “influences”. Clearly, 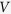 is additionally affected by 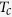, which varies independently (different thermal inertiasand paces) of 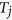 in transient states. During transient states, the small magnitude of outgoing Planck radiation reduces 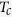 drastically. In turn, the decrease of 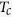 affects 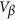 in an unknown sophisticated manner. At state 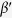, changes of 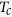 and 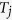 become synchronized again, as they did at state 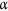. In other words, in the remedial approach (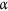 → 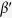), data between two end states are intentionally ignored. Because of dismissing 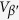 value, we need to produce another equation in substitution. Consequently, the next task is to obtain a relationship between 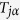 and 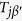 based on the principle of thermal anchoring. Following the first law of thermodynamics, we identify all energy components crossing the boundary of the sample’s control volume (Fig. 1a), and write 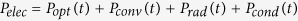 (Supplementary S4). Finally, from state 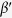 to state 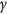, we are allowed to utilize the steady-state 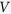 & 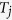 relationship, which is approximately linear with a negative slope. If time between 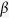 and 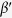 is taken to be 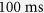, we obtain 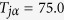 °C for B1# sample (Supplementary S5). Since it remains uncertain to precisely locate the state 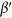, next we propose a previously-unreported method that adopts the principle of thermal anchoring and avoids PISO. In the steady-state Shockley equation for diodes, namely,


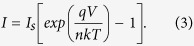


Since 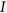, 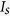 and 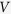 can be readily measured via experiments, we have only the ideality factor 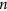 and 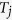 left as unknowns, and need one more equation.

In analogy to casting the anchor when docking a ship in the harbor so that the anchor location reveals ships’ whereabouts, we maintain 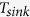 constant and attempt to determine 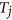. The overall thermal resistance of two layers, namely the die attach, and Cu slug, between the LED chip and the sink resembles the length of the anchoring steel wire.

In reference to the physical configuration of the sample (Fig. 1a), it is reasonable to idealize these layers as one-dimensional slabs. Consider a multi-layer system whose top and bottom are either heat sinks or sources. We further recognize the phenomenon that, when phonon waves propagate from the source to the sink, they excite oscillations of lattice inertia along the path, but do not alter basic lattice structures after they pass. When they reach the sink, 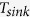 remains constant, but vibration energy escapes to outside the sink, and thermal conductivities of intermediate layers remain unchanged. If the electrical input 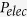 also remains unchanged (implying 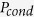 remains the same), so does 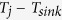. Then, we select two states, 1 and 2, where 1 represents 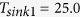 °C; 2 denotes 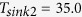 °C (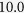 degrees higher than 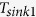. Other 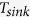 differences ranging from 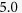
°C to 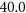 °C with a 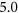 °C increment have also been conducted). Under the iso-current condition (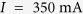), we observe that 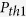 equals 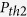 (because 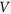 varies minimally) and that 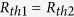 (for example, 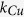 varies from 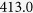 to 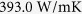 when its temperature varies from 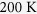 to 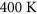)^32^. Therefore, we can safely deduce 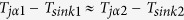 (Fig. 4a,b). As a result, we obtain two nonlinear relations,


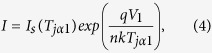


and


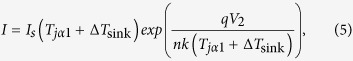


where 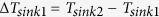. Equations (4) and (5) can be simultaneously solved using the Newton-Raphson method^33^ or its modified version (Supplementary S6). Values of 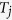 agree well with those obtained using CRS, TC and TI (Fig. 4c–e). Additionally, we have found this 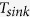 difference of 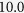 °C to be optimal among other 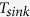 differences. If 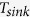 becomes too large, 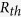 no longer remains constant, violating the nonlinear thermal anchoring principle. If 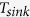 becomes too small, two algebraic equations tend to be similar, leading to algebraic redundancy.

Steps of the procedure can be outlined as:

- Measure the reverse current versus the junction temperature to obtain 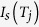.
- Measure the 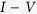 characteristic curve from 1 mA to 500 mA at 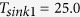 °C and 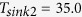 °C, respectively
- To solve equations (4) and (5) using Newton-Raphson method to obtain 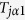 at 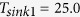 °C.

In summary, the discrepancy between PISO and non-PISO is attributed to non-synchronization of lattice and carrier temperatures in transient states. Generally in PISO carrier transient behaviors are intentionally bypassed, rendering the voltage and the carrier’s temperature disengaged. To confirm and avoid this PISO-induced disengagement, we first discover the LITA mechanism and develop an original, accurate, and nondestructive technique to measure LED junction temperatures in steady state conditions. This principle of the nondestructive method involves
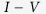
characteristic of LEDs and nonlinear thermal anchoring. Finally, NTA results exhibit close agreements with data of Raman spectroscopy, thermal couples, and thermal imagers (Fig. 4c–e, Fig. S3a,b).

## Methods

### Forward voltage method

**Figure 2:**
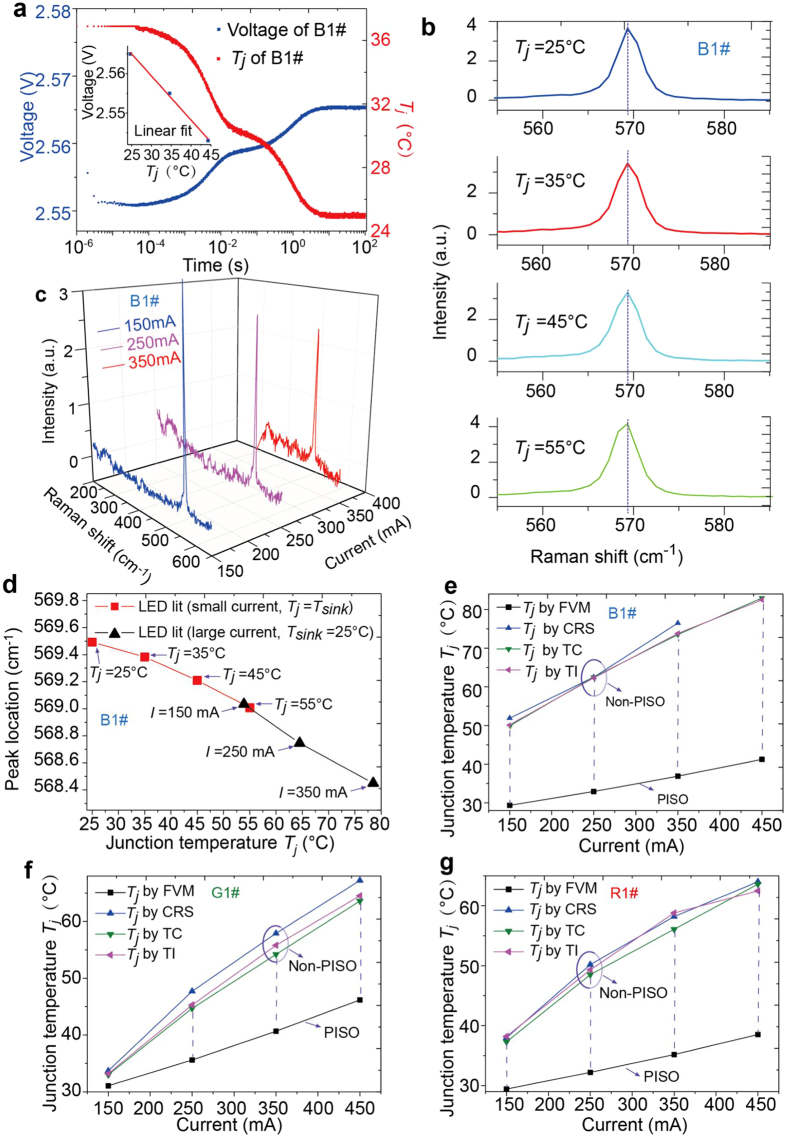
Junction-temperature measurements of FVM, CRS, TC and TI. (**a**) Take blue InGaN/GaN LED (B1#) as the example. In reference to the linear relationship between 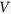 and 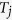, we deduce the value of 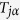 to be 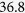 °C. (**b**) Relationship between 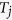 and Raman redshift for the B1# sample when the LED chip is lit at small currents (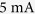). The peak at 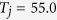 °C has shifted to the left slightly. (**c)** Relationship between 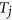 and Raman redshift for B1# sample when the LED chip is lit at large currents. At steady state and 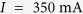, for example, we measure Raman shift to obtain the peak location. Next, utilizing the 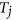 and Raman shift relationship in **b**, we obtain 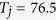 °C. (**d)** Correlation between peak location and 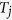. (**e)** Junction temperature versus the current for B1# sample. In the absence of PISO, results obtained by CRS, TC and TI agree closely with one another, but differ appreciably from those obtained by FVM. Due to the disturbance of large noises, 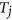 cannot be reliably measured in CRS for B1# sample at 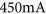. (**f**) Junction temperature versus the current for G1# sample. (**g**) Junction temperature versus the current for R1# sample.

**Figure 3:**
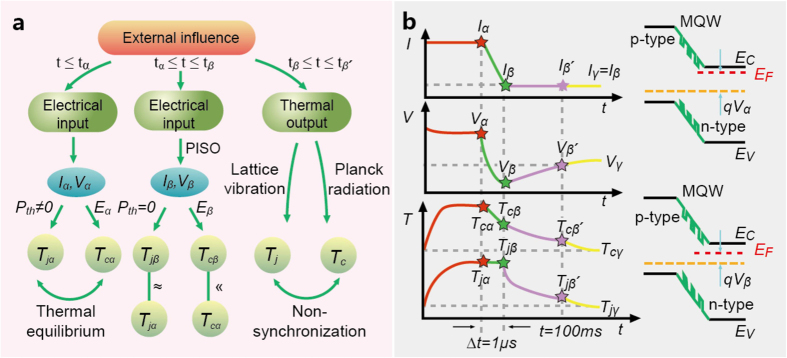
Microscopic system schematic explaining lattice-inertia thermal anchoring (LITA). (**a**) Non-synchronization between 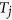 and 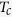 from state 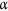 to state 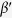. (**b**) Time evolutions of 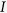, 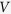 , and 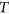 at various states: 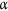, 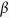, 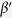, and 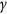. For 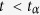, 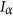 equals 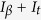. At 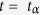, the switch for 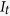 is suddenly turned off (PISO). For 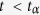, 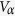 indicates the difference between 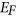 in the absence and the presence of external voltage. At 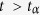, because the current has been switched down, 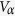 is reduced to 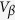. All samples contain multiple-quantum wells (MQW) to elevate illuminating efficiencies, as shown.

**Figure 4:**
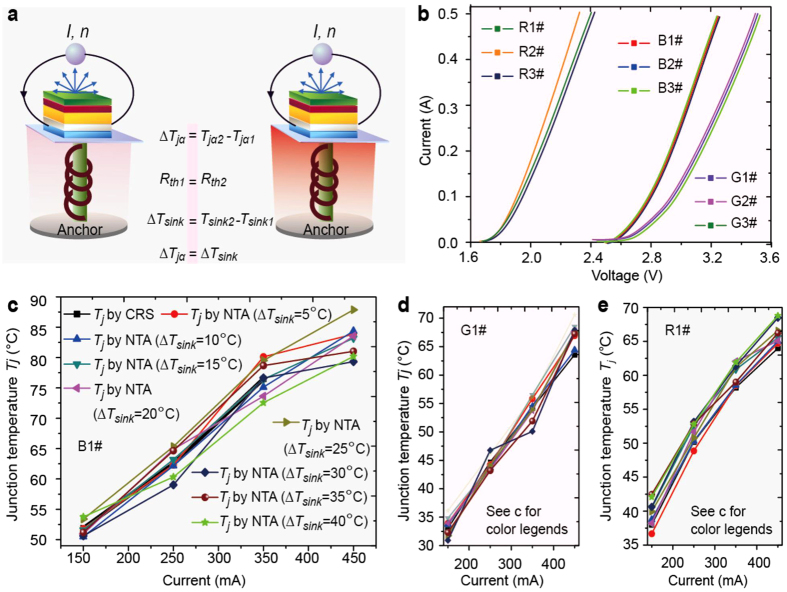
Schematic of nonlinear thermal-anchoring (NTA) and 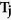 results. (a) In thermal anchoring, 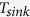 behaves as the anchor, which is maintained constant by the temperature controller, and can also transport the thermal energy away to outside the sample. The experimental procedure includes: (1) set 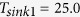 °C, and obtain I-V characteristic curves of nine LED samples for various currents ranging from 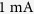 to 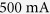. Measurements are taken 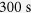 after the current is switched on, assuring that the steady state was reached. (2) set 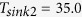 °C and measure I-V characteristic curves as step (1). (**b**) IV characteristic curves measured according to the experimental procedure in **a** for nine samples at 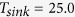 °C. These curves for same-colored samples appear almost indistinguishable. (**c)** 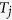 measured using NTA for B1# sample. (**d**) 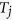 measured using NTA for G1# sample. (e) T j measured using NTA for R1# sample. NTA for G1# sample. (**e**) 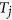 measured using NTA for R1# sample.

FVM includes three primary steps: (a) obtain a steady-state linear relationship between the voltage and 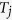 (inset of Fig. 2a), (b) operate PISO from state 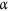 to state 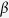, and (c) allow the sample to cool down from state 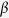 to state 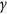 (steady state) (Fig. 2a). Take blue InGaN/GaN LED (B1#) as the example. At three sink temperatures (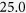 °C, 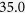 °C, and 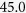 °C) and 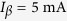, we measure three different voltages (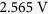, 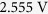, and 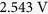), and obtain a negative-sloped line representing the relationship between 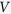 and 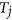, with 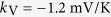 (inset). Next, we run a steady state current at 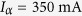 for 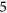 minutes. Instantaneously, the current is switched down to 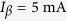 with the duration lasting approximately 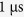. At this instant, the voltage, 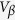, is recorded. After approximately 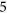 more minutes, the voltage is recorded to be 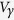. In reference to the linear relationship between 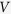 and 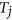, we deduce the value of 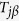, according to 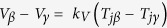, to be 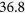 °C, which is assumed to equal 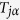 in FVM.

### Confocal Raman spectroscopy

CRS includes two primary steps: (a) measure Raman shifts for various 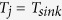 values to obtain a relationship between Raman shift and 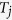 when LED is lit at small currents (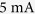). The LED sample is mounted on a heat sink, controlled by a temperature controller (Keithley Instruments, Inc., American, Keithley 2510), and is lit by a current source (Keithley Instruments, Inc., American, Keithley 2611). (b) measure Raman shifts to obtain desired 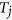 when LED is litby large currents. Raman shift signals are collected by a confocal Raman microscope (XploRA, HORIBA Jobin Yvon, France) to yield a correlation between the wave-peak location and the junction temperature when the LED is lit at small currents (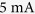). After the acquisition of this shift and 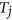 relationship, we turn on the LED at large currents and measure the Stokes shift again. Because the B1 chip emits 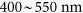 light beams, we select the 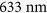 laser, carefully maintain all parametric conditions the same as the small-current run at 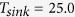 °C, and measure Raman shifts under currents of 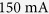, 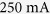, and 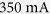.

### Thermocouples

TCs are placed on the sample surface for several random positions and take average values (Supplementary S1).

### Thermal imager

TI aims at the chips surface and takes the average of measurements distributed within a pre-determined area (Supplementary Fig. S1b–d).

### Nonlinear thermal-anchoring

NTA principle combined with Shockley equation generates two nonlinear equations which are solved by Newton-Raphson method. All first-order derivatives are discretized using the finite difference method, with the occasional necessary to adopt the under-relaxation algorithm to achieve convergences.

## Additional Information

**How to cite this article**: Zhang, J. *et al*. Non-synchronization of lattice and carrier temperatures in light-emitting diodes. *Sci. Rep.* **6**, 19539; doi: 10.1038/srep19539 (2016).

## Supplementary Material

Supplementary Information
