# Supplementary material for: Non-synchronization of lattice and carrier temperatures in light-emitting diodes
Source: Sci Rep. 2016 Jan 20;6:19539. doi: 10.1038/srep19539 (PMC4726174; doi:10.1038/srep19539)
Supplement: Supplementary Information [file srep19539-s1.pdf]

Supplementary to
"Non-synchronization of lattice and carrier temperatures in light-emitting diodes"

S1. Measurements of junction temperatures using thermocouples (TC) and a thermal imager (TI)

Thermocouples (TES-1310 K-type Nickel-chromium alloy and nickel silicon alloy) were used to measure temperatures of exposed LED surfaces (Fig. S1a). The diameter of the thermocouple tip was measured by a vernier caliper to be 0.2 mm, significantly smaller than the area of LED chip (1 mm $\times$ 1 mm). Several measurements weretaken at random positions on the chip surface to yield the mean value. In addition, a thermal imager, capable of providing non-intrusive measurements, was used to record the infrared (IR) imagery (25 fps) of the chip surface (Fig. S1b-d), with the distance between the sample and the camera lens being precisely the focal point of camera lens. When the LED sample was unlit, T_{sink} was set to be 343.0 K. At steady state, the surface temperature was observed and recorded to be the same, allowing the sample emissivity to be determined as 0.68. When the chip was lit to measure T_j for four different currents, this very emissivity value was used.

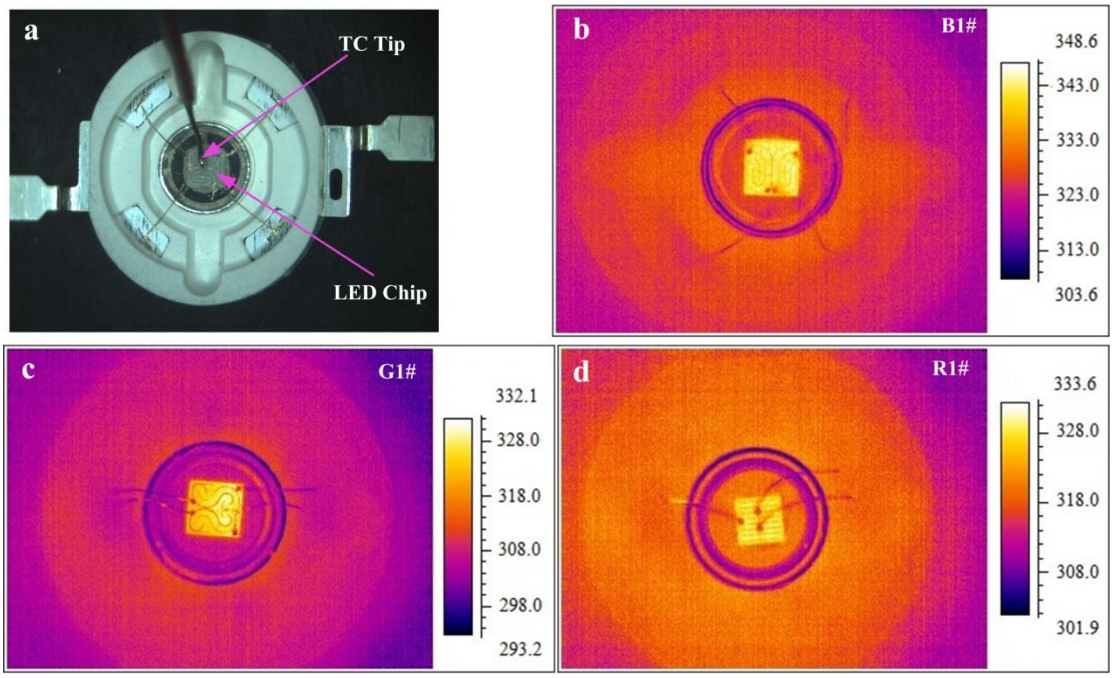

Figure S1 | Experimental results obtained by thermocouple (TC) and thermal imager (TI). a, The TC tip is placed on the LED chip to measure surface temperatures. The photo shows the comparison between sizes of the TC junction tip and the LED chip. Conspicuously, the former is smaller than the latter, thus ensuring sufficient resolutions. **b,** The infrared imagery obtained by TI for B1# sample. **c,** The infrared imagery obtained by TI for G1# sample. **d,** The infrared imagery obtained by TI for R1# sample.

S2. Detailed results of junction temperature measurements for other samples

Raman shifts signals are collected by a confocal Raman microscope for G1# sample and R1# sample. Because both the G1# chip and R1# chip emit visible light beams, we select the785 nm laser to measure Raman shifts under currents of 150mA, 250mA, 350 mA, and 450mA. All junction temperatures obtained by CRS, NTA (details in the main text), TC and TI agree closely with one another, but differ appreciably from those obtained by FVM.

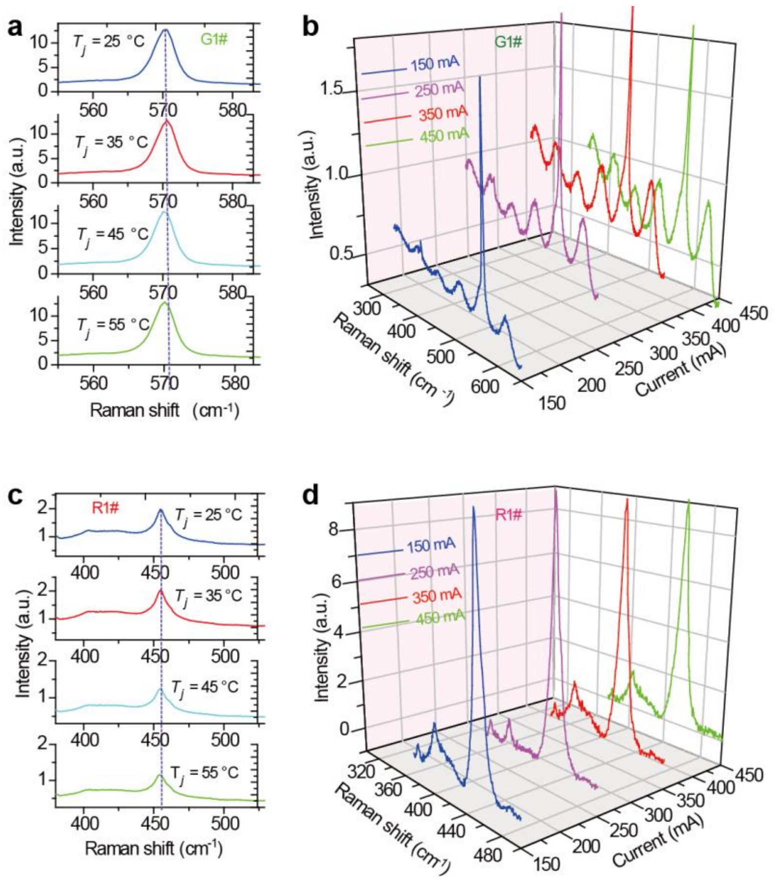

Figure S2 | Experimental results obtained by confocal Raman spectroscopy for G1# sample and R1# sample. a, Relationship between T_j and Raman redshift for the G1# sample when the LED chip is lit at small currents (5mA).**b,** Relationship

between T_j and Raman redshift for G1# sample when the LED chip is lit at large currents. **c,** Relationship between T_j and Raman redshift for the R1# sample when the LED chip is lit at small currents (5mA). **d,** Relationship between T_j and Raman redshift for R1# sample when the LED chip is lit at large currents.

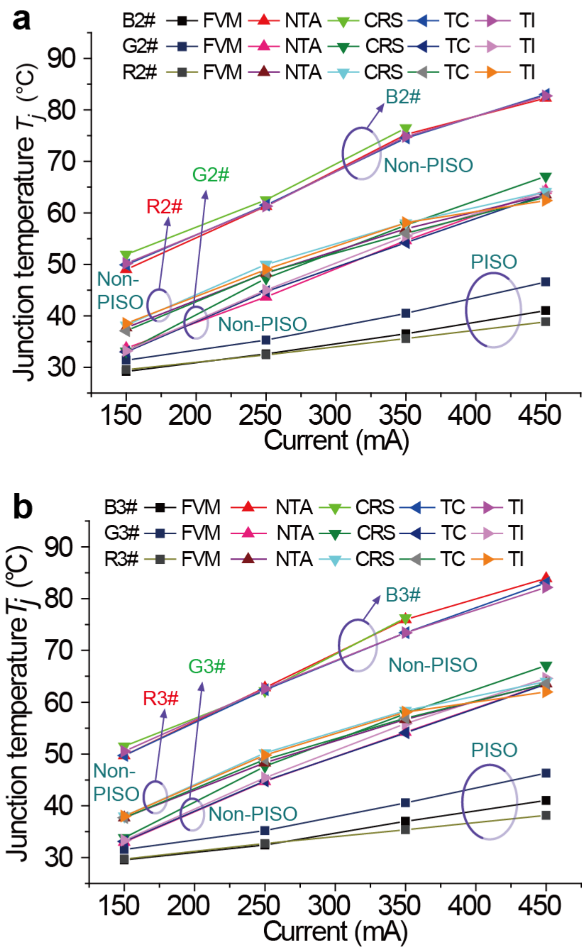

Figure S3 | Junction temperature results. a, Junction temperature versus the current for B2#, G2# and R2#. **b,** Junction temperature versus the current for B3#, G3# and R3#.

S3. Proof that the electron velocity at steady state α is higher than that at state β (after PISO)

In the vector form, the electron transport velocity in state α equals the sum of the thermal velocity and the drift velocity, i. e.,

$$\overrightarrow{v_\alpha} = \overrightarrow{v_{t\alpha}} + \overrightarrow{v_{d\alpha}},$$

$$\overrightarrow{v_\beta} = \overrightarrow{v_{t\beta}} + \overrightarrow{v_{d\beta}}, \quad \text{(S-1)}$$

or in reference to Fig. S4 and under the assumption that the drift direction is horizontal,

$$\overrightarrow{v_\alpha} = (|v_{d\alpha}| + |v_{t\alpha}| \cos\theta_\alpha)\vec{\imath} + (|v_{t\alpha}| \sin\theta_\alpha)\vec{\jmath},$$

$$\overrightarrow{v_\beta} = (|v_{d\beta}| + |v_{t\beta}| \cos\theta_\beta)\vec{\imath} + (|v_{t\beta}| \sin\theta_\beta)\vec{\jmath}, \quad \text{(S-2)}$$

leading to

$$|v_\alpha|^2 = |v_{d\alpha}|^2 + |v_{t\alpha}|^2 + 2|v_{d\alpha}||v_{t\alpha}| \cos\theta_\alpha,$$

$$|v_\beta|^2 = |v_{d\beta}|^2 + |v_{t\beta}|^2 + 2|v_{d\beta}||v_{t\beta}| \cos\theta_\beta, \quad \text{(S-3)}$$

or

$$|v_\alpha|^2 - |v_\beta|^2 = (|v_{d\alpha}|^2 - |v_{d\beta}|^2) + (|v_{t\alpha}|^2 - |v_{t\beta}|^2) + (2|v_{d\alpha}||v_{t\alpha}| \cos\theta_\alpha - 2|v_{d\beta}||v_{t\beta}| \cos\theta_\beta). \quad \text{(S-4)}$$

Since (1) $|v_{d\alpha}|^2 > |v_{d\beta}|^2$ due to PISO, (2) $|v_{t\alpha}|^2 - |v_{t\beta}|^2 \geq 0$, and (3) $\cos\theta\alpha$ and $\cos\theta_\beta$ are random numbers between -1 and 1, statistically yielding zero average values of $\cos\theta\alpha$ and $\cos\theta_\beta$, we conclude that $v_\alpha^2 > v_\beta^2$.

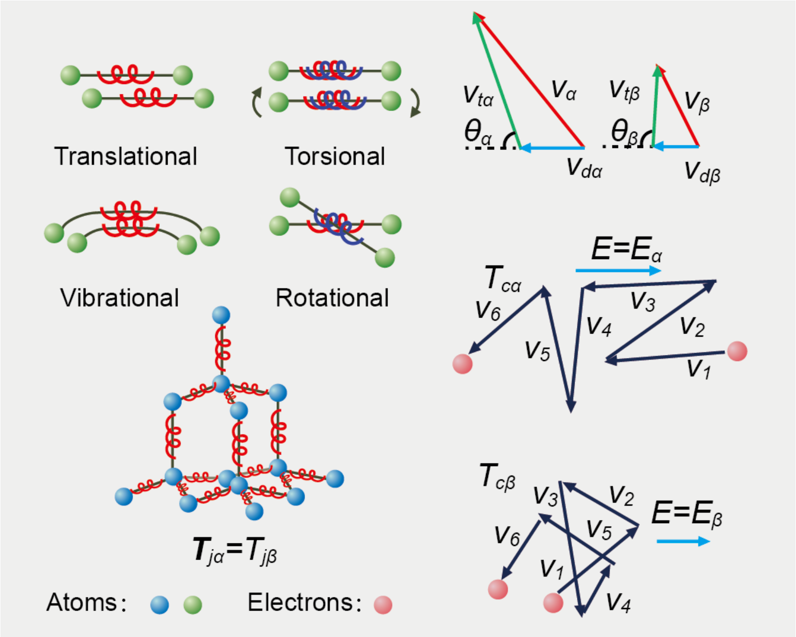

Figure S4 | Two different statuses of lattice inertia and carrier velocities. Junction temperatures are represented by lattice translation, torsion, vibration, and rotation. The junction temperature is approximately the same prior to and after PISO. By contrast, the velocity of an electron (v_α) at $t = t_\alpha$ prior to PISO is the vector sum of the thermally-diffusive velocity ($v_{t\alpha}$) and the drift velocity ($v_{d\alpha}$). During six time steps, forexample, this electron travels against the electrical field (E_α) prior to PISO and E_β after PISO.

S4. Justification of conductive thermal power being much greater than convection thermal power and thermal radiation

In reference to Fig. 1a, we write

$$P_{conv} = Ah(T_j - T_\infty) \approx 10^{-6} \times 10 \times (76.5 - 25.0) = 5.15 \times 10^{-4}\text{W}, \quad \text{(S-5)}$$

and

$$P_{rad} = A\varepsilon\sigma(T_j^4 - T_{inf}^4) = 2.71 \times 10^{-4}\text{W}, \quad \text{(S-6)}$$

where subscripts "conv", "rad", "j", "∞" and "surr" stand for "convection", "radiation", "junction", "ambience" and "surroundings"; A the surface of the chip; h the heat transfer coefficient; ε the emissivity of LED chip surface, σ the Stefan-Boltzmann constant. Consequently, we observe that $P_{conv} + P_{rad} = 7.86 \times 10^{-4}$ W $\ll$ 0.8 W, suggesting that the electrical power input minus the emitted light, $(P_{elec} - P_{opt})$, is transformed into P_{cond}.

S5. Cooling of the lump-capacitance model

At steady state, the energy conservation over the sample can be written as

$$P_{cond} = (T_{j\alpha} - T_{sink})/R_{th}, \quad \text{(S-7)}$$

where R_{th} denotes the overall thermal resistance between the chip and the heat sink. Therefore, $R_{th} = (T_{j\alpha} - T_{sink})/P_{cond}$. In the transient state, the first law of thermodynamics dictates that

$$mc_v\left(T_j - T_j^p\right) = -\Delta t(T_j - T_{sink})/R_{th}. \quad \text{(S-8)}$$

After algebraic rearrangement, we obtain

$$T_j = (c_1 T_{sink} + T_j^p)/(1 + c_1), \quad \text{(S-9)}$$

where $c_1 = (\Delta t P_{cond})/(mc_v(T_{j\alpha} - T_{sink}))$, and $T_{j\alpha}$ remains as an unknown. Using a MATLAB code, we compute T_j for $t \in [0, t_{\beta'}]$ (Fig. S5). Iterations are carried out to determine T_j to yield values in the inset table. Qualitatively, we can observe that $T_{j\alpha}$ values approach those obtained using CRS, TC and TI, suggesting that a remedy can be made if we allow a period of relaxation time based on the thermal anchoring principle (see the main text).

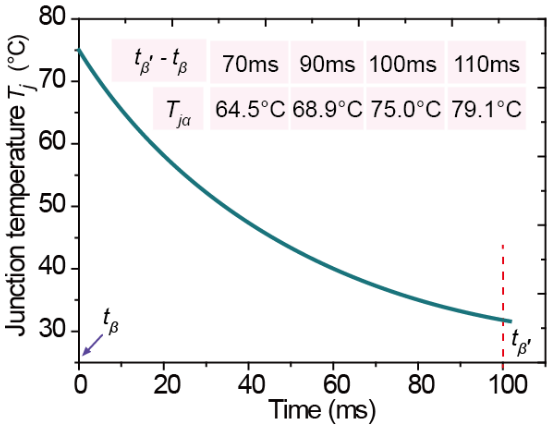

Figure S5 | Junction temperature versus time for B1# sample.

S6. Nonlinear thermal anchoring (NTA) method

1. Avoiding coexistence of exponential terms of large magnitude and I_s of small magnitude in normalized Shockley equation (SE)

Typical sets of theoretical values, for small values of $I_s(< 10^{-8}\text{A})$ and large exponential values $exp[V\ /\ (nkT_j)]$ $(\geq 10^6)$, satisfy SE. During experiments, however, measurement inaccuracies may be aptly introduced when these two extreme values are multiplied. For remedying purposes, a set of reference values are first introduced. Under small currents (1 mA), $T_j = T_{jo} = T_{sink} = T_{surroundings} = 298.0\ \text{K}$ is chosen. Let $I^* = I/I_o$, $I_s^*(T_j) = I_s(T_j)/I_{so}(T_{jo})$, $V^* = V/V_o$, and $\eta = q/k$ be introduced. The final non-dimensionalized SE can be derived as

$$I^* = I_s^*(T_j)exp\left[\eta V_o\left(\frac{V^*}{nT_j} - \frac{1}{n_oT_{jo}}\right)\right] = c_1 I_s^*(T_j)exp\left(\frac{\eta V}{nT_j}\right), \qquad \text{(S-10)}$$

where $c_1 = exp\left[-\eta V_o/\left(n_o T_{jo}\right)\right]$. Then the referenced ideality factor, n_o, can be computed [Refs. 1~4]. Equation (S-10) is preferable to Eq. (3) in the main text since, in the former, extreme values have vanished.

2. Reverse current and I-V characteristic curve

Equation (S-10) contains $I_s^*(T_j)$, which needs to be determined prior to its usage. When LED is unlit, six T_j values were taken within a range of typical LED sink temperatures from $298.0\ \text{K}$ to $348.0\ \text{K}$ in $10.0\ \text{K}$ increments. The $I_s$values were measured with a $-5\ \text{V}$ (most commonly used) bias voltage. All measurements were conducted in pitch darkness, using 4-wire Kelvin probes. For convenience, we can further curve-fit these measurements with a correlation, $I_s^*(T_j) = \beta_1\, exp\left(-T_j/\beta_2\right) +$

β_3, all values of β_1, β_2 and β_3 are listed in Table S1. In Fig.S6, all dimensionless I_s^* are normalized on their values at 298.0 K.

Table S1 Parametric values in the relationship between $I_s^*(T_j)$ and T_j

LED sample	β_1	β_2	β_3
**B1#**	-2.85×10^3	36.40	1.80
**B2#**	-5.51×10	95.07	3.37
**B3#**	-5.05×10^2	61.71	5.10
**G1#**	-3.19×10^{12}	10.38	2.09
**G2#**	-2.11×10^{14}	9.24	3.06
**G3#**	-2.54×10^{14}	9.16	2.89
**R1#**	1.13×10^{-3}	-59.81	0.83
**R2#**	8.06×10^{-4}	-57.76	0.86
**R3#**	1.92×10^{-3}	-58.60	0.69

The I-V characteristic curves of nine LED samples were obtained from1 mA to 500 mA at $T_{sink} = 298.0$ K (Fig. 4b in the main text). Measurements were taken 300 s after the current was switched on, assuring that the steady state was reached.

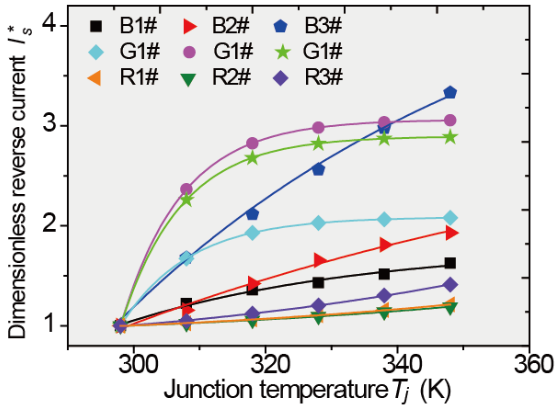

Figure S6 | Dimensionless reverse saturation current, I_s^*, versus junction temperature for nine different samples. As T_j increases, I_s^* increases.

3. Ideality factor for small currents

Values of the ideality factor n for small currents are obtained below. Equation (2) in the main text can be rearranged into

$$lnI = lnI_s + \frac{\eta V}{nT_j}. \tag{S-11}$$

Since I_s is independent of the forward voltage, V, differentiating Eq. (A-11) with respect to V leads to $\partial(lnI)/\partial V = \eta/nT_j$, or

$$n = \frac{\eta}{T_j}\left(\frac{\partial V}{\partial lnI}\right). \tag{S-12}$$

Next, the IV characteristic curve of the LED sample was measured when the sample was driven by small currents (1 μA $\sim$ 10 mA) with T_{sink} maintained at 298.0 K. For the sake of minimizing the self-heating, the current is turned on for 10 ms and off for 200 ms intermittently. The voltage isrecorded during the on phase using the 4-wire Kelvin probe. The ideality factor n_o (Fig. S7) is computed based on Eq. (S-12). From Fig. S4, the ideality factor n_o is valid only within the region where V is linear with lnI (dashed line on the Fig.S4) [See Ref.1]. All values are: 1.4 of B1#, 1.5 of B2#, 1.5 of B3#,4.2 of G1#, 4.6 of G2#, 4.5 of G3#, 1.4 of R1#, 1.3 of R2#, and 1.3 of R3#, respectively.

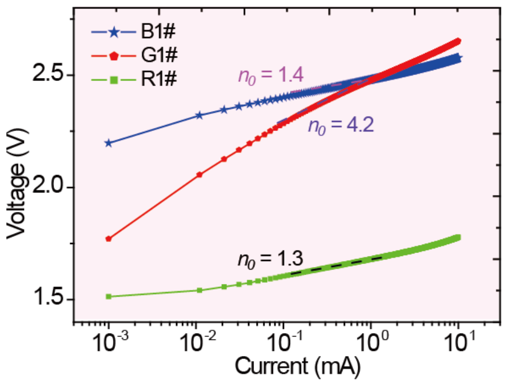

Figure S7 | The referenced ideality factors n_o for B1#, G1# and R1#.

4. Serial electrical resistance

A diode is inevitably accompanied by a parasitic serial resistance, R_s. Rigorously, Eq. (3) in the main text needs to be modified into

$$I = I_s exp\left(\frac{\eta(V-IR_s)}{nT_j}\right), \quad \text{(S-13)}$$

which can be differentiated with respect to I to yield

$$1 = I_s exp\left(\frac{\eta(V-IR_s)}{nT_j}\right)\frac{\partial(V-IR_s)}{\partial I}, \quad \text{(S-14)}$$

or

$$R_s = \frac{dV}{dI} - \frac{nT_j}{\eta I}, \quad \text{(S-15)}$$

whose values for B1# sample are: $1.4\ \Omega$ of 150 mA, $1.2\ \Omega$ of 250 mA, $1.0\ \Omega$ of 350 mA, and $0.9\ \Omega$ of 450 mA,respectively.

If the voltage of idealized LED, namely $V_{id} = V - IR_s$, is used (instead of V) to obtain $T_{j,id}$ (the junction temperatures of idealized LED), these $T_{j,id}$ values will deviate appreciably from experimental counterparts. In reality, R_s behaves as a heat source whose thermal energy is supplied and transformed from the electrical power

($I^2 R_s$). As a result, it tends to increase the $T_{j,id}$ value. In other words, the voltage of a realistic LED should represent the combination of idealized LED and IR_s. Furthermore, during experimental studies of LED performances, it is difficult to single out the $T_{j,id}$ measurement due to the integrated nature of the LED physical structure. These $T_{j,id}$ values obtained by using V_{id} values disagree with those obtained by experiments.

References

1. Zhu, D. *et al.* The origin of the high diode-ideality factors in GaInN/GaN multiple quantum well light-emitting diodes. *Appl. Phys. Lett.* **93**, 253107 (2008).
2. Lee, J. M. & Kim, S. B. Analysis of current components and estimation of internal quantum efficiency in light-emitting diodes. *IEEE Trans. Electron Devices* **58**, 3053-3057 (2011).
3. Shah, J. M., Li, Y. L., Gessmann, T. & Schubert, E. F. Experimental analysis and theoretical model for anomalously high ideality factors ($n \geq 2.0$) in AlGaN/GaN p-n junction diodes. *J. Appl. Phys.* **94**, 2627-2630 (2003).
4. Dai, Q.*et al.* Internal quantum efficiency and nonradiative recombination coefficient of GaInN/GaN multiple quantum wells with different dislocation densities. *Appl. Phys. Lett.* **94**, 111109 (2009).

S7. Miscellaneous unofficial proofs of possible flaws in FVM

Out of curiosity, when two of authors touched the LED sample surface with their fingers, it was definite that they felt sharp burning pains due to the heat conduction transferred from LED to their fingers, suggesting that the surface could not have possibly been at 36.8 °C.

To confirm(or question) the validity of FVM, the first author made an intentional trip to Chengdu, Sichuan Province, China, to meet and consult the chief engineer of Mentor Graphics. His answer can be summarized as that the burning discomfort felt by her finger was caused by the radiation emitted by the LED sample. However, how could the radiation emitted by a light source at 36.8 °C (if T_j was, indeed, 36.8 °C) possibly generate a burning discomfort to her skin whose temperature is 36.0 °C?

In a separate trip, she also visited National Testing Center for LED Application Products (NTCLAP), and took measurements of junction temperatures for samples (that were same nine samples described in the main text) using the standard T3ster purchased by NTCLAP (identical model type used at Xiamen University). Results include $T_j = 28.9$ °C for B1# sample at 150 mA, $T_j = 32.1$ °C at 250 mA, $T_j = 36.3$ °C at 350 mA, and $T_j = 41.2$ °C at 450 mA, in close agreements with those shown in Fig. 2e, suggesting that we have used the functional instrument and have followed correct procedures required for FVM.
